# Lamin A and Prelamin A Counteract Migration of Osteosarcoma Cells

**DOI:** 10.3390/cells9030774

**Published:** 2020-03-22

**Authors:** Camilla Evangelisti, Francesca Paganelli, Gaia Giuntini, Elisabetta Mattioli, Alessandra Cappellini, Giulia Ramazzotti, Irene Faenza, Maria Cristina Maltarello, Alberto M. Martelli, Katia Scotlandi, Francesca Chiarini, Giovanna Lattanzi

**Affiliations:** 1 CNR Institute of Molecular Genetics “Luigi Luca Cavalli-Sforza” Unit of Bologna, 40136 Bologna, Italy; camilla.evangelisti@cnr.it (C.E.); e.mattioli@area.bo.cnr.it (E.M.); 2 IRCCS Istituto Ortopedico Rizzoli, 40136 Bologna, Italy; 3 Department of Biomedical and Neuromotor Sciences, University of Bologna, 40126 Bologna, Italy; francesca.paganell16@unibo.it (F.P.); alessandra.cappellini@unibo.it (A.C.); giulia.ramazzotti@unibo.it (G.R.); irene.faenza2@unibo.it (I.F.); alberto.martelli@unibo.it (A.M.M.); 4 Department of Molecular and Development Medicine, Cellular and Molecular Physiology Unit, University of Siena, 53100 Siena, Italy; gaia.giuntini@student.unisi.it; 5 Laboratory of Musculoskeletal Cell Biology, IRCCS Istituto Ortopedico Rizzoli, 40136 Bologna, Italy; mariacristina.maltarello@ior.it; 6 Experimental Oncology Laboratory, IRCCS Istituto Ortopedico Rizzoli, 40136 Bologna, Italy; katia.scotlandi@ior.it

**Keywords:** osteosarcoma, lamin A, cellular migration, *LMNA* gene, osteoblast differentiation

## Abstract

A type lamins are fundamental components of the nuclear lamina. Changes in lamin A expression correlate with malignant transformation in several cancers. However, the role of lamin A has not been explored in osteosarcoma (OS). Here, we wanted to investigate the role of lamin A in normal osteoblasts (OBs) and OS cells. Thus, we studied the expression of lamin A/C in OS cells compared to OBs and evaluated the effects of lamin A overexpression in OS cell lines. We show that, while lamin A expression increases during osteoblast differentiation, all examined OS cell lines express lower lamin A levels relative to differentiated OBs. The condition of low *LMNA* expression confers to OS cells a significant increase in migration potential, while overexpression of lamin A reduces migration ability of OS cells. Moreover, overexpression of unprocessable prelamin A also reduces cell migration. In agreement with the latter finding, OS cells which accumulate the highest prelamin A levels upon inhibition of lamin A maturation by statins, had significantly reduced migration ability. Importantly, OS cells subjected to statin treatment underwent apoptotic cell death in a RAS-independent, lamin A-dependent manner. Our results show that pro-apoptotic effects of statins and statin inhibitory effect on OS cell migration are comparable to those obtained by prelamin A accumulation and further suggest that modulation of lamin A expression and post-translational processing can be a tool to decrease migration potential in OS cells.

## 1. Introduction

Osteosarcoma (OS) is the most common primary bone tumor in children and adolescents and therefore has an important social impact despite its rarity [1]. OS displays a high degree of aggressiveness and tendency to metastasize [2]. Surgical resection combined with chemotherapy is the most effective therapeutic strategy against OS [3] and this multidisciplinary approach has improved the survival of patients with localized tumors over the past few decades, achieving a 5-year survival rate of up to 70%. However, the prognosis for patients with metastasis at diagnosis or for those who do not respond to first-line treatments remains poor [3,4]. The numerous and complex genetic aberrations which characterize OS have slowed down the identification of specific common oncogenic drivers of the disease and the identification of more efficient therapeutic strategies, especially for those patients who present with metastases [2,5].

The transforming events leading to OS development occur in multipotent mesenchymal stem cells (MSCs) and/or osteoblast progenitors in any phase of differentiation [6]. Transformation induces a block in physiological development, associated with an abnormal proliferation processes, and loss of cell differentiation, which is a common biological aspect in OS, with strong implications in predicting tumor aggressiveness [7,8]. Thus, restoring differentiation seems to be an attractive strategy to be exploited for therapeutic purposes.

Several studies provided evidence that tumorigenic potential and malignant transformation may be related to modulation of nuclear lamins [9,10,11,12].

Lamins are key components of the nuclear lamina that provide shape, integrity and rigidity to the nucleus. Importantly, lamins interact with chromatin and chromatin-binding partners, including regulators of cellular proliferation and importantly differentiation [13]. The different roles of lamins in cellular processes have made these proteins the topic of debate for their role in cancer progression [13]. This led to the conclusion that lamins contribute to tumorigenesis and progression.

Altered lamin expression in tumors may increase nuclear deformability and could favor the ability of cells to transit tight interstitial spaces, promoting metastasis [14,15]. Therefore, lamin alterations could support tumor cells in escaping the physiological control of proliferation and death program.

Decreased expression of lamin A has been detected in small cell lung cancer and it has also been reported in adenocarcinoma of stomach, colon and esophageal carcinoma [10]. Furthermore, reduced or negative lamin A expression is associated with poor prognosis in a number of cancers, including gastric carcinoma, lymphomas, lung, colon and breast cancers [16,17,18,19,20]. It has also been observed that loss of lamin A correlates with disease progression, metastasis and poor prognosis in patients with diffuse large B-cell lymphoma and breast cancer [21,22,23].

However, the role of lamin A/C has not been explored in OS. Here, we focused on investigating lamin A/C relevance in several OS cell lines. We first studied the expression of lamin A/C in OS compared to osteoblasts (OBs) and evaluated the effects of lamin A overexpression in OS cell lines. Our results show that all OS cell lines have lower lamin A/C expression as compared to non-transformed differentiated OBs. Low lamin A levels are related to higher cellular proliferation and migration abilities.

Prelamin A, the precursor of lamin A, is known to play a critical role in chromatin organization and transcriptional regulation [24,25]. Inhibition of lamin A maturation by statins elicits accumulation of prelamin A [24]. Here, we show that overexpression of unprocessable prelamin A or statin treatment reduces OS migration. These results indicate that modulation of lamin A expression or post-translational processing can be exploited to decrease migration potential in OS.

## 2. Materials and Methods

### 2.1. Cell Cultures, Transfection and Treatments

The patient-derived human osteosarcoma cell lines HOS, 143B, MG63, MOS, SaOS2, U2OS were obtained from Deutsche Sammlung von Mikroorganismen und Zellkulturen GmbH (DSMZ, Braunschweig, Germany), while IOR-MOS, OS9, OS10 and OS20 were established in Dr. Scotlandi’s laboratory [26]. All OS cell lines were cultured in DMEM (1 mg/mL glucose, Gibco, Thermo Fisher Scientific, Waltham, MA, USA), supplemented with 10% fetal bovine serum (FBS, Life Technologies, Monza, Italy), 2mM l-glutamine, 100 U/mL penicillin and 100 μg/mL streptomycin (Sigma-Aldrich, Saint Louis, MO, USA) at 37 °C in a humidified atmosphere of 5% CO_2_.

Adipose-derived Stem Cells (ADSCs) were purchased from Thermo Fisher Scientific and cultured in Complete MesenPro RS medium (Gibco, Thermo Fisher Scientific, Waltham, MA, USA), in a 37 °C incubator with 5% CO_2_, as described in the manufacturer’s protocol. ADSCs were differentiated toward the osteogenic lineage by seeding 5 × 10^3^ cells/cm^2^ and replacing the culture medium after 2 days with Complete STEMPRO Osteogenesis Differentiation Medium (Gibco, Thermo Fisher Scientific, Waltham, MA, USA) [27].

Primary osteoblasts isolated from healthy donors were obtained from BioLaM biobank (IOR CE approval 0018259-01-13, issued on 05/09/2016) and maintained under standard cell culture conditions of 5% CO_2_ and 95% humidity and were used at passages 3–5. For differentiation experiments, primary osteoblasts were cultured with osteogenic medium containing 10% FBS, 1% penicillin and streptomycin, 50 μg/mL of ascorbic acid, and 10 mM β-glycerophosphate. The medium was changed every 3 days.

For Alizarin Red Staining, cells were fixed with neutral buffered formalin (10%) for 10 min. The fixed cells were rinsed with PBS and stained with 0.5% Alizarin Red staining solution (Sigma-Aldrich, St. Louis, MO, USA) (pH 4.2) for 10 min at room temperature. After being washed with ddH_2_O on a shaking platform, the mineralized nodules were imaged by a scanner.

To overexpress Lamin A, 143B or HOS cells were transfected with pEmpty-EGFP (GFP) and pLamin A-EGFP plasmids (lamin A/GFP) (Clontech, Mountain View, CA, USA). Transient transfections were performed according to manufacturer’s protocol, using Lipofectamine 3000 (Thermo Fisher Scientific, Waltham, MA, USA). Transfected cells were plated in 12-well tissue culture plates or in flasks (70% confluence). Biochemical and immunofluorescence analyses were performed 24, 48 and 72 h after transfection.

*LMNA* silencing in MG63 cell line, was done by employing siRNAs duplexes specific for human *LMNA* (siRNA *LMNA*) purchased from Thermo Fisher Scientific (Waltham, MA, USA). Scrambled duplexes were used as control (silencer select negative control: 4390843). An amount of 0.15 × 10^6^ MG63 cells was plated in 6-well plates. At approximately 50% confluence, cells were transfected with the annealed siRNA-*LMNA* and siRNA-scrambled (siRNA final concentration 10 nM) using lipofectamine 3000. The experiments were performed after 48 h from transfection.

Overexpression of unprocessable prelamin A (LA-C661M) was obtained as reported elsewhere [28]. Biochemical analyses were performed 24 h after transfection. Mevinolin (Sigma Aldrich, Saint Louis, MO, USA), an inhibitor of the hydroxymethyl-glutaryl-synthase that impairs farnesylation of prelamin A, was added to cell culture medium at 5 or 10 µM and cells were harvested after 24, 48 or 72 h.

### 2.2. Cell Proliferation

To assess cellular growth, cells were seeded in 6-wells in DMEM plus 10% FBS. After 24 h, increasing concentrations of mevinolin were added, and the cells were exposed to the drug for up to 72 h. Growth curves were generated by plotting direct count of cells. Viable cells were counted by a hemocytometer using 0.2% Trypan Blue.

To test the cytotoxic effects of mevinolin, OS cell lines were also cultured for 24, 48 and 72 h in the presence of the vehicle (DMSO 0.1%) or increasing drug concentrations, and cell viability was determined using a MTT cell proliferation kit (3-(4,5-Dimethylthythiazol-2-yl)-2,5-diphenyltetrazolium bromide) (Roche Diagnostics, Basel, Switzerland), according to manufacturer’s instructions.

### 2.3. Wound Healing Assay

Cells were seeded in 6-well plates and the wound healing assay was performed. A reproducible longitudinal scratch in the monolayer was made the following day using sterile micropipette tips. The process of wound closure was monitored at 0 and 24 h by photographing the central field of the scratches under an inverted light microscopy (Olympus CKX41, Olympus Corp, Tokyo, Japan) mounted with a digital camera (C-7070 Wide Zoom, Olympus) at 10× magnification. The pictured field was standardized each time against a horizontal line drawn on the base of the plate passing through the center of each well. Morphometric analysis of cell migration was performed using a computerized image analysis system (Qwin, 3.0 software, Leica Microsystem Imaging Solution, Ltd., Wetzlar, Germany). A region that included the artificial scratch and the adjacent cell monolayer was selected as the standard region of interest (ROI). The wound healing effect was calculated as (1-Ax/A0) %, where A0 and Ax represented the empty scratch area at 0 and 24 h, respectively.

### 2.4. Annexin V-FITC/PI Staining

Apoptosis analysis was determined through the Annexin V-FITC Apoptosis Detection kit (eBioscience, Thermo Fisher Scientific, Waltham, MA, USA), according to the manufacturer’s instructions, using an FC500 flow cytometer (Beckman Coulter, Brea, CA, USA) equipped with the appropriate software (version 2.2, CXP, Beckman Coulter). At least 10.000 events were acquired.

### 2.5. Western Blot Analysis

Western blot analysis was performed by standard methods. Briefly, cells were lysed in RIPA lysis buffer (containing 20 mM Tris-HCl (pH 7.5), 150 mM NaCl, 1 mM Na2EDTA, 1 mM EGTA1, % NP-40, 1% sodium deoxycholate, 2.5 mM sodium pyrophosphate, 1 mM, b-glycerophosphate 1 mM, Na3VO4, 0.1% SDS) and protease inhibitors. After sonication, protein fractions were collected by centrifugation at 15,000× *g*, 4 °C, for 10 min. A total of 20 μg of proteins were separated by SDS– PAGE using Criterion TGX polyacrylamide gels (Bio-Rad, Hercules, CA, USA) and blotted onto a nitrocellulose membrane (Bio-Rad, Hercules, CA, USA). Proteins were detected using the Amersham ECL Prime Western Blotting Detection Reagent (GEHealthcare, Little Chalfont, Buckinghamshire, England), the ChemiDoc-It2 Imaging System and the Vision Works LS Software for the analysis (UVP, LLC, Upland, CA, USA). Bands were revealed by the Amersham ECL detection system. The expression of specific proteins was tested by using the following antibodies: anti-osterix (OSX) (sc-393325 from Santa Cruz Biotechnology, Heidelberg, Germany) 1:500, anti-osteocalcin (sc-365797, Santa Cruz Biotechnology, Heidelberg, Germany) 1:500, anti-ROCK2 (sc-398519, Santa Cruz Biotechnology, Heidelberg, Germany) 1:800, anti-prelamin A (Merck Millipore, Burlington, MASS, USA #MABT858) 1:800, anti-lamin A/C (#4777 Cell Signaling technology, Danvers, MA, USA) 1:1000, anti-Ras (#3339 Cell Signaling technology, Danvers, MA, USA) 1:1000, and anti-GAPDH (#5174 Cell Signaling technology, Danvers, MA, USA) 1:1000, anti-cleaved caspase 3 (#9664 Cell Signaling technology, Danvers, MA, USA) 1:1000, anti-PARP (#9532 Cell Signaling technology, Danvers, MA, USA) 1:1000, anti-MMP9 (#3852 Cell Signaling technology, Danvers, MA, USA) 1:1000 (Cell Signaling Technology, Danvers, MA, USA), anti-Cathepsin K 1:1000 (Abcam, Cambridge, UK).

### 2.6. qRT-PCR

Total RNA was extracted using the RNeasy Mini Kit (Qiagen, Venlo, The Netherlands), according to the manufacturer’s instructions, and 1 μg of total RNA was reverse transcribed using High-Capacity cDNA Reverse Transcription Kit (Thermo Fisher Scientific, Waltham, MA, USA). Gene expression was assessed using the TaqMan^®^ Gene Expression Master Mix, using the 7300 RT-PCR system (Applied Biosystems, Foster City, CA, USA). Results were normalized to the level of the ubiquitously expressed RNA 18S ribosomal 1 gene (*RN18S*), and were expressed as 2^−ΔCt^ (ΔCt = CT gene of interest − CT internal control) to compare the relative gene expression among samples, and as 2^−ΔΔCt^ (ΔΔCt = ((CT gene of interest − CT internal control) sample − (CT gene of interest − CT internal control) universal)) to compare gene expression of the treated cell lines with that of untreated control [29]. The alkaline phosphatase *(ALPL)* probe (Hs.PT.56a.40555206) was from Roche Diagnostics (Basel, Switzerland), and the *LMNA* probe (TaqMan Hs.01108900_g1) was from Thermo Fisher Scientific (Waltham, MA, USA). The *RUNX2* (Taqman Hs.00231692_m1) probe was from Thermo Fisher Scientific (Waltham, MA, USA).

### 2.7. Immunofluorescence Microscopy

To determine the subcellular localization of lamin A/C and prelamin A, OS cells were seeded in 12-well plates and the cells were grown on coverslips, fixed with methanol for 8 min at −20 °C. Cells were then blocked with 3% BSA-containing PBS for 1 h. Antibodies diluted in 3% BSA-containing PBS were applied overnight at 4 °C, and revealed by using FITC-conjugated secondary antibodies diluted 1:200 (incubated for 1 h at RT). Samples were mounted with a DAPI-containing anti-fade reagent (Molecular Probes, Thermo Fisher Scientific, Waltham, MA, USA) and observed with a Nikon Eclipse Ni epifluorescence microscope. The images captured with NIS-Elements 4.3 AR software were processed using Photoshop CS4 (Adobe Systems, Inc., San Jose, CA, USA). The following antibodies were used: anti-prelamin A (Merck Millipore, Burlington, MASS, USA), diluted 1:500, anti-lamin A/C (#4777 Cell Signaling Tecnhology, Danvers, MA, USA), diluted 1:100.

### 2.8. Statistical Analysis

Statistical analyses were performed using Student’s *t* test for *LMNA* overexpression/silencing experiments, for cell growth and apoptosis studies, and for migration experiments, while one-way ANOVA and Dunnett’s multiple comparison test was employed to evaluate differences in the expression levels of proteins or genes analyzed. Correlations were established according to Spearman’s rank test. At least three different experiments were performed and data plotted represent mean ± SD (standard deviation) with a significance level of *p < 0.05, **p < 0.01, ***p < 0.001 (GraphPad Prism Software, Version 5, San Diego, CA, USA).

## 3. Results

### 3.1. Lamin A/C Expression Increases in Differentiating Osteoblasts

**Figure 1:**
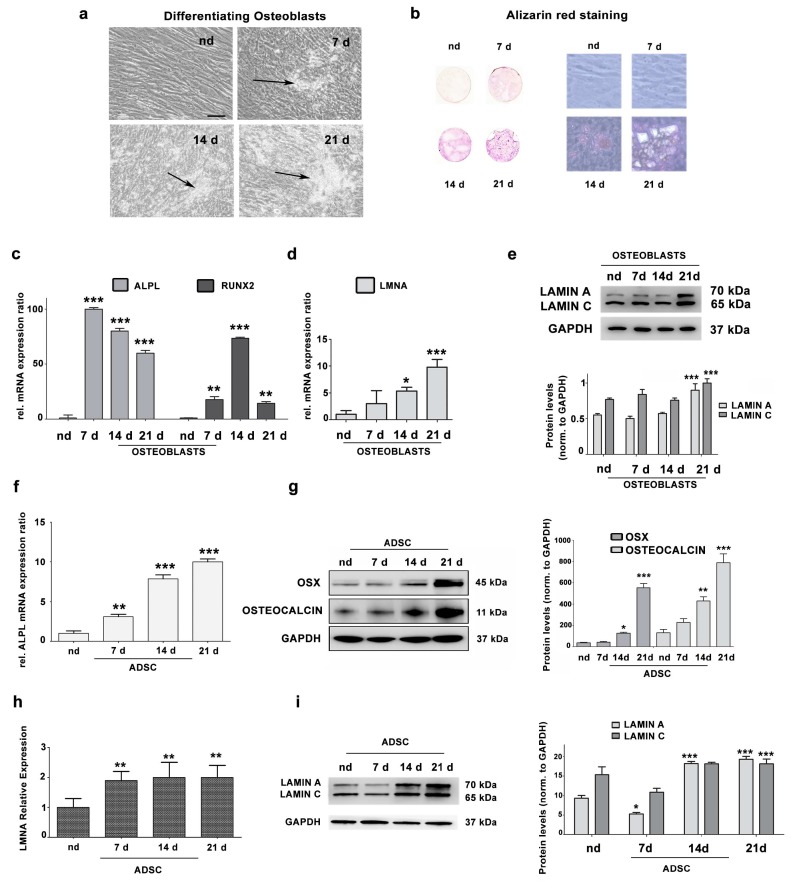
(**a**) Representative images of osteoblasts cultured for 7, 14, 21 days in differentiating medium. nd: cells cultured in DMEM plus 10% FBS for the same time. Arrows indicate hydroxyapatite crystals. Scale bar: 20 μm. (**b**) Alizarin Red staining of osteoblasts at 7, 14 or 21 days in differentiation medium. nd indicates non-differentiated osteoblasts from a healthy donor, cultured for 21 days in DMEM plus 10% FBS. Left pictures, alizarin red-stained cultures on coverslips imaged by a scanner; right pictures, phase contrast images (20× magnification) of the same cell cultures showing hydroxyapatite crystals. (**c**) Increased *ALPL* and *RUNX2* gene expression by qRT-PCR analysis. Statistical analyses were performed using Student’s *t* test and asterisks indicate statistically significant differences with respect to nd, **p < 0.01, ***p < 0.001. (**d**) *LMNA* gene expression analysis by qRT-PCR in nd versus differentiated osteoblasts (OBs). Asterisks indicate statistically significant differences with respect to nd, *p < 0.05, ***p < 0.001 (**e**) Western blotting analysis of lamin A/C protein expression in nd and differentiated OBs. Densitometric analysis is shown as mean values ± SD of three different experiments. Statistical analyses were performed using Student’s *t* test and asterisks indicate statistically significant differences with respect to nd, ***p < 0.001. (**f**) Increased *ALPL* gene expression by qRT-PCR analysis. Statistical analyses were performed using Student’s *t* test and asterisks indicate statistically significant differences with respect to non-differentiated adipose-derived stem cells (ADSCs) (nd), **p < 0.01, ***p < 0.001. (**g**) Protein expression analysis of Osterix (OSX) and Osteocalcin by western blotting. Densitometric analysis is reported, showing significantly increased levels of OSX and Osteocalcin compared to non-differentiated ADSCs (nd). Statistical analyses were performed using Student’s *t* test. Asterisks indicate statistically significant differences with respect to non-differentiated ADSCs (nd), **p < 0.01, ***p < 0.001. (**h**) *LMNA* gene expression analysis by qRT-PCR in ADSCs versus differentiated OBs. Asterisks indicate statistically significant differences with respect to non-differentiated ADSCs (nd), **p < 0.01. (**i**) Western blotting analysis of lamin A/C protein expression in non-differentiated ADSCs (nd) and differentiated OBs. Densitometric analysis is reported as mean ± SD. Asterisks indicate statistically significant differences with respect to non-differentiated ADSCs (nd), *p < 0.05, ***p < 0.001.

To evaluate lamin A/C expression in differentiating osteoblasts from healthy donors, we cultured cells in differentiation medium up to 21 days. Morphological changes and formation of hydroxyapatite crystals in cell cultures were observed starting from day 7 of culture in differentiation medium (Figure 1a). Osteoblast differentiation was confirmed by alizarin red staining (Figure 1b), as well as by expression levels of *ALPL* and *RUNX2* genes, well-established osteoblast differentiation markers, which were strongly upregulated, with a peak at day 7 and day 14, respectively (Figure 1c). The *LMNA* gene was significantly upregulated during osteoblast differentiation (Figure 1d). Moreover, lamin A/C protein levels were significantly increased in osteoblasts at 21 days of differentiation, relative to undifferentiated cells (nd) (Figure 1e).

These data were confirmed in adipose tissue-derived pluripotent stem cells (ADSCs), which can differentiate into different cell types, including osteoblasts [27,30]. Figure 1f shows that *ALPL* gene expression significantly increased in a time-dependent manner during ADSC osteogenic differentiation. Moreover, protein levels of osteogenic differentiation markers Osteoblast-specific Transcription Factor Osterix (OSX) and Osteocalcin were significantly increased in ADSCs at 14 and 21 days in differentiating medium (Figure 1g). During ADSC osteogenic differentiation, *LMNA* gene expression was significantly increased (Figure 1h). Moreover, the level of lamin A/C protein was decreased in ADSC cells at 7 days in osteogenic differentiation medium and increased at day 14 and 21 (Figure 1i).

### 3.2. Lamin A/C Expression is Reduced in Osteosarcoma Cells

**Figure 2:**
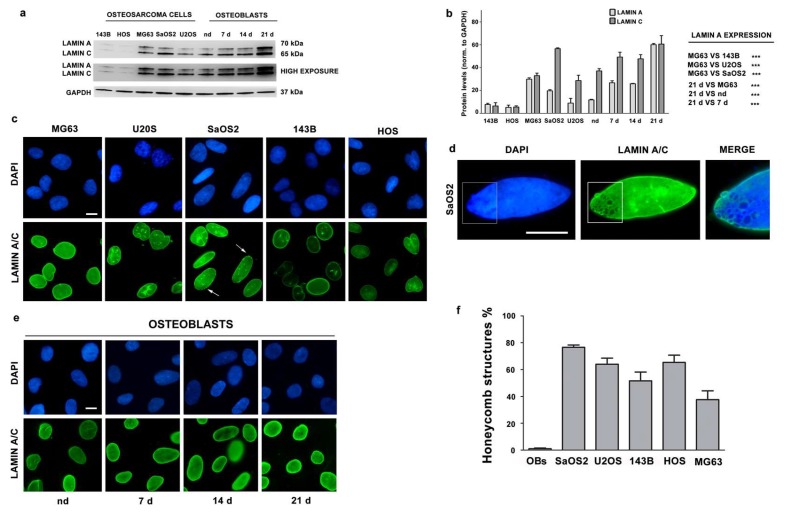
(**a**) Western blotting analysis of lamin A/C in osteosarcoma (OS) cell lines, and OBs from healthy donor differentiated for 7, 14, 21 days in osteogenic medium. nd indicates non-differentiated cells from a healthy donor, cultured 21 days in DMEM plus 10% FBS. (**b**) Densitometric analysis of lamin A/C as mean values ± SD of three different experiments. Asterisks indicate statistically significant differences with respect to 143B cells or nd cells, ***p < 0.001. (**c**) Immunofluorescence staining of lamin A/C in OS cell lines. Arrows indicate honeycomb structures. (**d**) High magnification of a honeycomb structure in a SaOS2 nucleus. The area indicated in the rectangle is shown at higher magnification in the merged image (merge). (**e**) Immunofluorescence staining of lamin A/C in osteoblasts (OBs) from healthy donors differentiated for 7, 14 or 21 days in osteogenic medium. (**f**) Percentage of honeycomb structures detected in osteoblasts at all differentiation stages or in osteosarcoma cells. Mean of values ± SD are plotted. Cells in c, d and e were labeled using anti-lamin A/C antibody and revealed by an AlexaFluor 488 secondary antibody (green). DNA was stained with DAPI (blue). Scale bars, 10 μm.

We evaluated lamin A/C protein levels in several OS cell lines by western blot analysis. Relative to OBs from healthy donors differentiated for 21 days, all OS cell lines displayed significantly lower lamin A expression levels (Figure 2a,b).

However, as compared to other OS cell lines, MG63 showed higher protein levels of lamin A (Figure 2a,b). The lowest lamin A/C levels were determined in HOS cells, and in the 143B Ras-mutated HOS-derived cell line [31,32] (Figure 2a,b). These data were also confirmed in other OS cell lines obtained in our laboratory (Supplementary Figure S1).

We have also evaluated lamin A/C localization and we found that lamin A/C localized at the nuclear periphery (Figure 2c). Morphological abnormalities were observed both in osteoblasts and osteosarcoma cells, although honeycomb structures, which are typical of some laminopathic cells, were only detected in OS nuclei (Figure 2c,f).

### 3.3. Migration and Proliferation Ability are Increased in OS Cells with Low LMNA Expression

**Figure 3:**
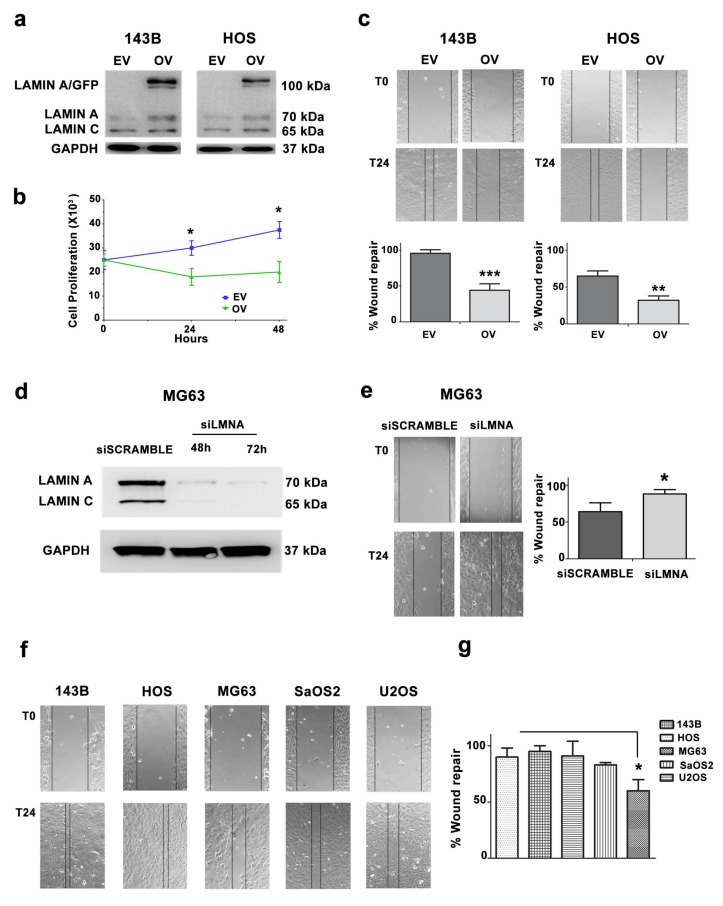
(**a**) Western blotting analysis of 143B and HOS cells transfected with GFP and lamin A/GFP. EV: empty vector cells, OV: cells overexpressing lamin A/GFP. (**b**) Cell growth of 143B-transfected cells (EV and OV) was assessed by staining cells with Trypan Blue and counting viable cells after 24 and 48 h after transfection. Asterisks indicate statistically significant differences with respect to EV-transfected cells, *p < 0.05. (**c**) Wound healing assay in 143B- and HOS-transfected cells (EV: empty vector, OV: cells overexpressing lamin A/GFP). Representative pictures were taken at 0 and 24 h after scratching. Magnification 10×. Histograms of cell migration of 143B- and HOS-transfected cells are plotted. Columns show the mean ± SD of three independent experiments. Asterisks indicate statistically significant differences with respect to EV-transfected cells, **p < 0.01, ***p < 0.001. (**d**) Western blotting analysis of siScramble- and si*LMNA*-transfected MG63 at 48 and 72 h of transfection. (**e**) Wound healing assay of siScramble- and si*LMNA*-transfected MG63. Representative pictures were taken at 0 and 24 h after scratching. Magnification 10×. Histograms were plotted as mean ± SD of three independent experiments. (**f**) Wound healing assay of OS cell lines. (**g**) Histograms of OS cell migration in wound healing assay as mean ± SD. Asterisks indicate statistically significant differences with respect to HOS cells, *p < 0.05.

To gain insight into the role of lamin A in OS cells, we used 143B and HOS cell lines, which have the lowest lamin A/C levels and we expressed a lamin A/GFP fusion protein. After 24 h of transfection, 143B and HOS cells expressed lamin A/GFP (Figure 3a).

Then, we wanted to analyze the effect of lamin A overexpression on cellular proliferation. Empty GFP and lamin A/GFP-transfected cells were seeded at the same density and cell growth was assessed by trypan blue exclusion assay and cell count. Overexpression of lamin A clearly slowed down proliferation of 143B cells 24 and 48 h after transfection with lamin A/GFP vector (Figure 3b).

Given that OS is a highly metastatic tumor and lamin A is involved in cellular migration [11], we tested migration potential of lamin A-overexpressing cells. Lamin A/GFP-transfected cells displayed a significantly lower ability to migrate in a wound healing assay as compared to cells only expressing GFP (Figure 3c). These results indicated that lamin A reduces the migratory ability in OS cells. To confirm the role of lamin A in cellular migration, we silenced the *LMNA* gene in MG63 cells and performed a wound healing assay, demonstrating that the silenced cells migrated faster compared to scramble (Figure 3d,e).

Based on the latter results, we performed a wound healing assay on five OS cell lines. 143B and HOS cells were able to almost completely repair the wound after 24 h. Of note, these cells expressed the lowest lamin A/C levels among tumor cell lines here examined. On the contrary, MG63 cells, expressing the highest lamin A amount among OS cell lines, failed to totally repair the wound in the same time frame (Figure 3f,g). Thus, OS cell lines with the highest lamin A levels showed significantly reduced wound healing ability, suggesting an inverse correlation between lamin A/C protein amount and cellular migration. We performed a Spearman’s Rank correlation test, obtaining an r value = −0.7 (*p* < 0.05). This confirmed the existence of an inverse correlation between lamin A expression levels and cellular migration ability in the OS samples analyzed.

### 3.4. Prelamin A Accumulation Reduces Migration Potential of OS Cells

**Figure 4:**
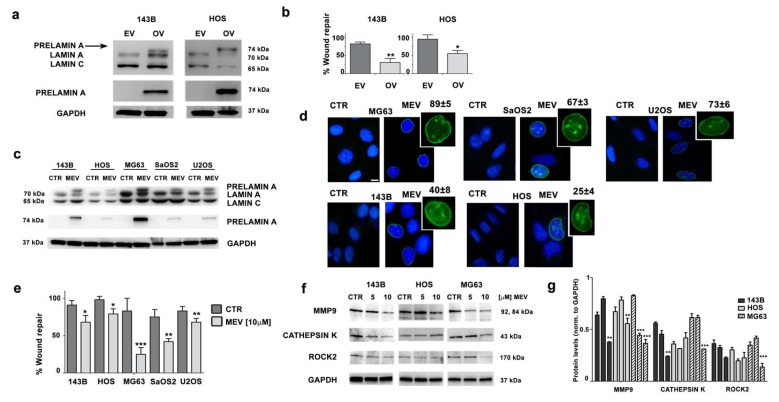
(**a**) Western blotting analysis of 143B and HOS cells overexpressing prelamin A. EV, cells transfected with empty vector; OV, cells overexpressing unprocessable prelamin A (LA-C661M). Prelamin A expression was assessed using a lamin A/C antibody which detects lamin A, lamin C and prelamin A. An anti-prelamin A antibody, which recognizes only prelamin A, was also employed. (**b**) Wound healing assay performed in 143B and HOS cells overexpressing prelamin A. Results are the mean ± SD of three independent experiments. Asterisks indicate statistically significant differences versus empty vector cells (EV), *p < 0.05, **p < 0.01. (**c**) Western blotting analysis of OS cell lines treated with mevinolin (10 µM) for 24 h. Lamin A/C and prelamin A were detected by anti-lamin A/C or anti-prelamin A antibodies. CTR: untreated cells; MEV: cells treated with 10 µM mevinolin. (**d**) Immunofluorescence analysis of prelamin A in OS cells treated with mevinolin (10 µM) for 24 h. Cells were labeled using anti-prelamin A antibody revealed by an AlexaFluor488 secondary antibody. Scale bar 10 µm. The percentage of prelamin A-positive cells in each cell line is reported. (**e**) Wound healing assay of OS cell lines treated with mevinolin (10 µM) for 16 h. Results are the mean ± SD of three different experiments. Asterisks indicate statistically significant differences versus untreated (CTR) cells, *p < 0.05, **p < 0.01, ***p < 0.001. (**f**) Western blotting analysis of MMP9, Cathepsin K, and ROCK2 proteins in 143B, HOS, and MG63 cells treated with mevinolin for 48 h. (**g**) Densitometric analysis is shown as mean ±SD values of three different experiments. Asterisks indicate statistically significant differences with respect to CTR cells, **p < 0.01, ***p < 0.001.

We then wondered if an unbalance in lamin A maturation could reduce migration potential of OS cells. To test this hypothesis, we expressed an unprocessable prelamin A sequence (LA-C661M), which causes accumulation of non-farnesylated prelamin A, in 143B and HOS cells, which are the OS cell lines with the lowest lamin A expression. Western blotting analysis showed prelamin A accumulation in both cell lines (Figure 4a). Accumulation of prelamin A induced a strong decrease in migration ability in 143B and HOS cells (Figure 4b).

Mevinolin is a drug known to reduce production of farnesyl-pyrophosphate. As a consequence, mevinolin blocks prelamin A farnesylation, which is required for further protein processing, thus impairing the whole process of lamin A maturation and causing accumulation of non-farnesylated prelamin A [33,34]. OS cell lines were treated with mevinolin and accumulation of prelamin A was obtained, as shown by western blot analysis (Figure 4c). Immunofluorescence staining of prelamin A confirmed this result and further showed typical nuclear morphological changes associated with prelamin A accumulation [24,28,35], consisting of intranuclear clusters and invaginations (Figure 4d). As expected, treatment with mevinolin caused a reduction in migration ability in all OS cells. This effect was mostly evident in MG63 cells, which accumulate the highest prelamin A amount, while milder effects of statins on cellular migration were observed in the other cell lines (Figure 4e).

Consistent with these observations, signaling effectors of cellular migration and invasion, like MMP9 (matrix metallopeptidase 9) and Cathepsin K, were decreased in statin-treated MG63 OS cells and, to a lesser extent in the other OS cell lines (Figure 4f,g). Finally, ROCK2 (Rho-associated protein kinase 2) kinase, which is a crucial intracellular mediator regulating osteosarcoma migration [36,37], was significantly downmodulated in MG63 OS cells (Figure 4f,g).

These findings indicated a major role for prelamin A in reducing cellular migration upon mevinolin administration.

### 3.5. Mevinolin Effects in OS Cells Are Related to Prelamin A Accumulation

**Figure 5:**
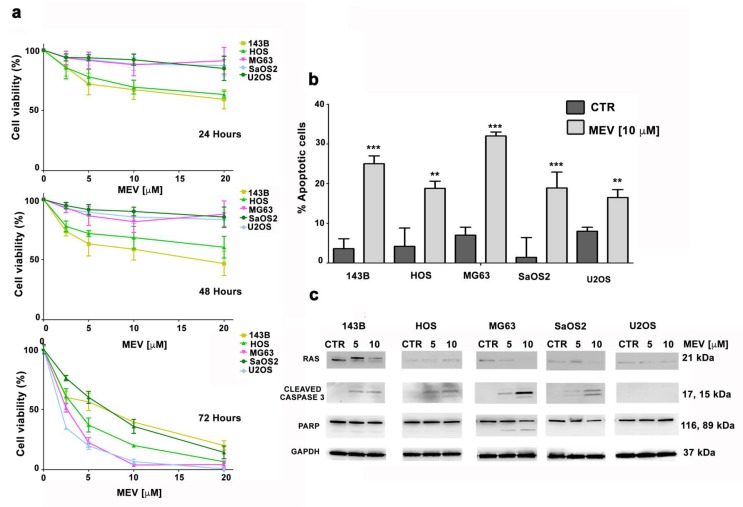
(**a**) MTT assays were performed at 24, 48, and 72 h after treatment with increasing concentrations of mevinolin. At least three independent experiments were performed. (**b**) OS cells were treated with 10 μM of mevinolin for 48 h and an Annexin V-FITC/PI staining assay was performed. The mean ± SD of three separate experiments is shown. CTR: untreated cells. Asterisks indicate statistically significant differences versus untreated (CTR) cells, **p < 0.01, ***p < 0.001. (**c**) Western blotting analysis of OS cell lines treated with mevinolin (5–10 µM) for 48 h.

Concentration-dependent viability was assessed upon mevinolin treatment in OS cells. After 24 or 48 h of treatment, only 143B and HOS cells showed a significant decrease in cell viability (around 25% when mevinolin was employed at 10 µM concentration) (Figure 5a).

Interestingly, although 72 h mevinolin treatment reduced cell viability in all OS cell lines, the most significant effect of mevinolin was observed in MG63 cells, expressing the highest prelamin A levels (Figure 5a). Moreover, after long-term mevinolin treatment, the highest increase in the percentage of apoptotic cells was observed in MG63 cell culture (Figure 5b).

It is well-known that cellular proliferation and survival, especially in tumor cells, is also related to Ras, whose activity is dependent on farnesylation [38]. Since 143B cells carry a Ras mutation [31,32] we reasoned that mevinolin-induced reduction of cellular viability could be also related to Ras de-farnesylation. Interestingly, while impairment of Ras farnesylation by mevinolin was evident in 143B cells, the highest induction of apoptotic markers, including cleaved Caspase 3 and PARP (Poly (ADP-ribose) polymerase), was observed in mevinolin-treated MG63 cells (Figure 5c). These results suggest that, although effects of mevinolin on cellular viability and apoptosis are in part due to Ras inhibition, the most efficient reduction of cell survival is associated with prelamin A accumulation.

## 4. Discussion

Although an increasing number of reports recently implicated lamins in human cancers, their role in OS, the most common primary malignant neoplasia of the skeletal system [39], has been elusive.

It was previously reported that lamin A is upregulated during OB differentiation of human bone marrow stromal cells, suggesting a critical role in osteogenesis [40]. Here, we firstly examined lamin A physiological expression in ADSCs and in differentiated OBs derived from ADSC cells or from healthy donors. Our findings revealed that lamin A expression directly correlates with the acquisition of differentiated status, as demonstrated in many cell types [41]. Further, our results are consistent with previous evidence demonstrating that lamin A/C knockdown suppresses OB differentiation [40,42].

In agreement with these considerations is also the finding that all OS cells here examined show lower lamin A expression levels relative to differentiated OBs. On the other hand, this observation also suggests that lamin A loss could play a role in OS tumorigenesis. Consistent with this hypothesis, we were able to show that elevated lamin A levels directly correlate with reduced migration and proliferation of OS cells and overexpression of lamin A in 143B OS cells, which express very low lamin A levels, is sufficient to significantly diminish cellular migration.

Based on these results, we tested the effects of mevinolin, a statin that interferes with processing of prelamin A, the lamin A precursor protein, which undergoes a complex post-translational processing requiring farnesylation of its *C*-terminus end [24]. Mevinolin inhibits HMG-CoA (3-hydroxy-3-methylglutaryl-coenzyme A) reductase by competitively binding a portion of the HMG-CoA binding site, thereby blocking the synthesis of isoprenoid lipids in the mevalonate pathway [43]. This condition reduces availability of the farnesyl moiety to many cellular constituents, among which prelamin A and Ras are examples. Statins have been extensively tested in humans, as they are widely used in hypercholesterolemia treatment. Moreover, previous observations in human and murine OS cells showed that statins sensitize OS cells to anticancer drugs and synergistically act with chemotherapeutic agents to reduce cell invasiveness [44,45]. Here, we focused on the role of prelamin A in statin-dependent effects. Although we confirm that mevinolin effects are related to Ras de-farnesylation and inhibition, our data show that the highest efficacy of mevinolin treatment is obtained in OS cells that accumulate the highest prelamin A levels. This supports the view previously suggested in a study performed in laminopathic mice [46], that prelamin A accumulation per se reduces tumor cell migration and metastatic potential. A correlation between accumulation of prelamin A and reduced cancer cell migration has also been demonstrated in pancreatic cancer cells using diverse drugs that inhibit lamin A maturation [47]. In addition, our results show that the first effect of statins in OS cells is to reduce cellular migration, while effects on cellular viability appear later on. This is not unexpected, provided the largely demonstrated mutual relationship between lamin A and prelamin A accumulation at the nuclear lamina and changes in cytoskeleton-dependent mechanisms [33,48,49].

Low lamin A expression could cause an increase of cellular migration due to effects of lamin A absence on mechanosignaling, as recently demonstrated [50]. The reason why prelamin A accumulation reduces OS cell migration deserves further investigation. A possibility could be that changes in *LMNA* levels affect linking of nucleoskeleton and cytoskeleton (LINC) proteins and LINC-dependent effects on mechanosignaling and cell migration. It is known that a prelamin A increase elicits high SUN1 levels [33,51,52]. This could impact on nucleo-cytoskeleton interplay. For instance, a recently published paper shows that low SUN1 levels favor migration of bone-marrow derived mesenchymal stem cells [53].

Although further studies in OS patient cohorts are necessary to validate the clinical relevance of our observations, the data presented here support for the first time a role of lamin A in the regulation of proliferation, differentiation and migration capabilities of OS cells and support the possible use of statins as adjuvant agent in the treatment of OS patients.

## Supplementary Materials

The following are available online at https://www.mdpi.com/2073-4409/9/3/774/s1, Figure S1: Lamin A/C Expression is Reduced in Osteosarcoma Cell Lines.
