# Supplementary figures and images for: Lamin A and Prelamin A Counteract Migration of Osteosarcoma Cells
Source: Cells. 2020 Mar 22;9(3):774. doi: 10.3390/cells9030774 (PMC7140691; doi:10.3390/cells9030774)

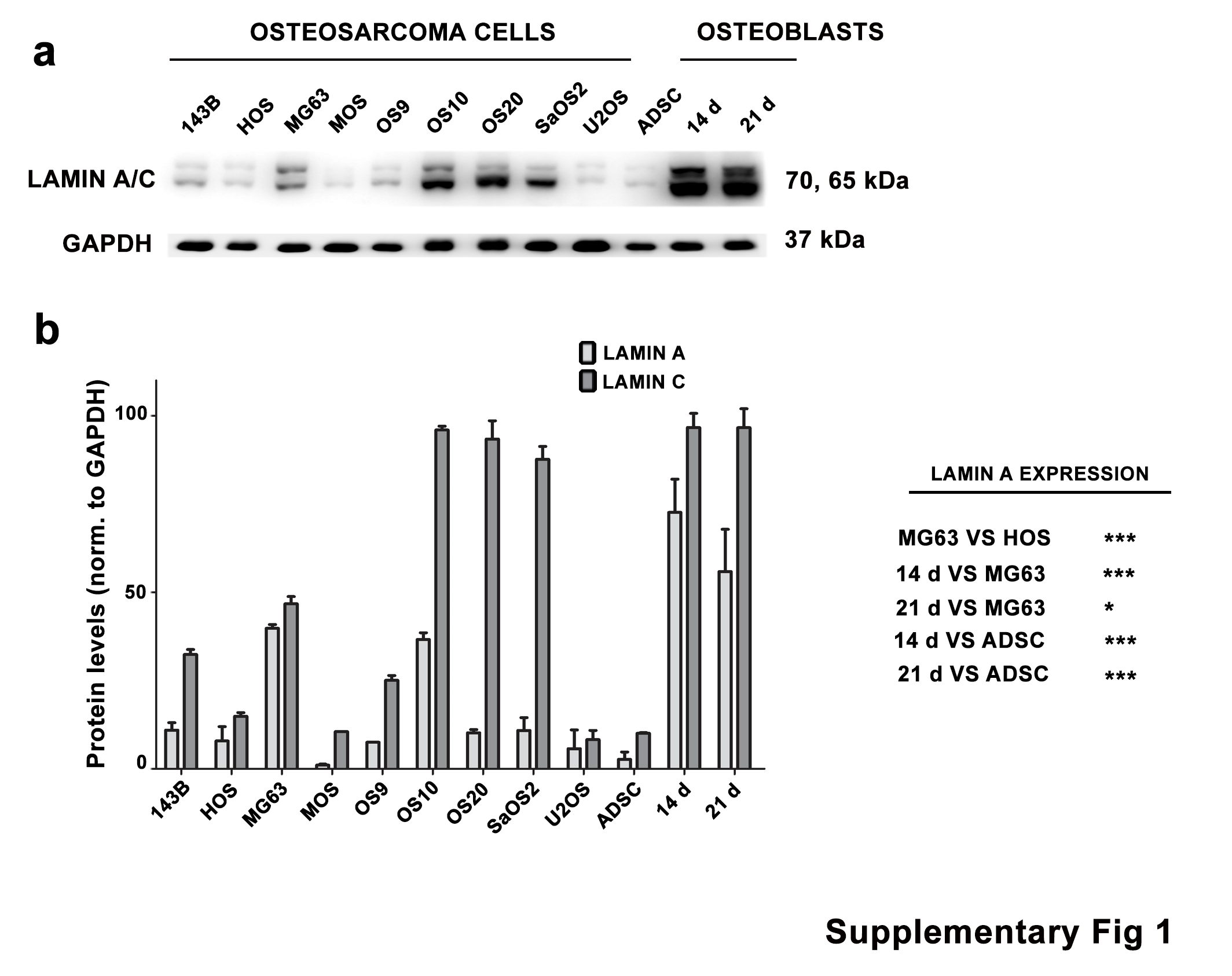

Supplement: Supplementary file 1 [file cells-09-00774-s001.zip › cells-699974-supplementary.tif]
